# A highly accurate heuristic algorithm for the haplotype assembly problem

**DOI:** 10.1186/1471-2164-14-S2-S2

**Published:** 2013-02-15

**Authors:** Fei Deng, Wenjuan Cui, Lusheng Wang

**Affiliations:** 1 Department of Computer Science, City University of Hong Kong, Kowloon, Hong Kong

## Abstract

**Background:**

Single nucleotide polymorphisms (SNPs) are the most common form of genetic variation in human DNA. The sequence of SNPs in each of the two copies of a given chromosome in a diploid organism is referred to as a haplotype. Haplotype information has many applications such as gene disease diagnoses, drug design, etc. The haplotype assembly problem is defined as follows: Given a set of fragments sequenced from the two copies of a chromosome of a single individual, and their locations in the chromosome, which can be pre-determined by aligning the fragments to a reference DNA sequence, the goal here is to reconstruct two haplotypes (*h*_1_, *h*_2_) from the input fragments. Existing algorithms do not work well when the error rate of fragments is high. Here we design an algorithm that can give accurate solutions, even if the error rate of fragments is high.

**Results:**

We first give a dynamic programming algorithm that can give exact solutions to the haplotype assembly problem. The time complexity of the algorithm is *O*(*n *× 2*^t ^*× *t*), where *n *is the number of SNPs, and *t *is the maximum coverage of a SNP site. The algorithm is slow when *t *is large. To solve the problem when *t *is large, we further propose a heuristic algorithm on the basis of the dynamic programming algorithm. Experiments show that our heuristic algorithm can give very accurate solutions.

**Conclusions:**

We have tested our algorithm on a set of benchmark datasets. Experiments show that our algorithm can give very accurate solutions. It outperforms most of the existing programs when the error rate of the input fragments is high.

## Background

The recognition of genetic variations is an important topic in bioinformatics. Single nucleotide polymorphisms (SNPs) are the most common form of genetic variation in human DNA. Humans are diploid organisms. There are two copies of each chromosome (except the sex chromosomes), one from each parent. The sequence of SNPs in a given chromosome copy is referred to as a *haplotype*. Haplotype information is useful in many applications, such as gene disease diagnoses [1,2], drug design, etc. Due to their essential importance in many biological analysis, haplotypes have been attracting great attention in recent years [3-7]. Since experimental methods for direct sequencing of haplotypes are both expensive and time consuming, computational methods are usually much more promising.

Currently, computational methods for computing haplotypes often fall into two categories: *population haplotyping *[8-11] and *haplotype assembly *(also known as *single individual haplotyping*) [12-15]. The former tries to compute haplotypes based on the genotype data from a sample of individuals in a population. Many software packages have been published in this field, e.g., PHASE [10]. An obvious drawback of population haplotyping lies in its weakness in recognizing rare and novel SNPs [16]. Contrary to population haplotyping, haplotype assembly is more efficient and has received more attention in recent years. The input to the haplotype assembly problem is a set of fragments sequenced from the two copies of a chromosome of a single individual, and their locations in the chromosome, which can be pre-determined by aligning the fragments to a reference DNA sequence. The task here is to reconstruct two haplotypes from the input fragments. In this paper, we focus on the haplotype assembly problem.

The haplotype assembly problem was first introduced by Lancia *et al. *[17]. In [17], the authors proposed three optimization criteria for solving this problem, i.e. *minimum fragment removal *(MFR), *minimum SNP removal *(MSR) and *longest haplotype reconstruction *(LHR). Some polynomial time algorithms have been designed to solve some versions of such optimization problems [18,19]. Lippert *et al. *[19] summarized the models in [17] and proposed some new models. Among these models, the most difficult and realistic one is *minimum error correction *(MEC), where we want to minimize the total number of conflicts (errors) between the fragments and the constructed haplotypes (*h*_1_, *h*_2_). The haplotype assembly problem with MEC is NP-hard [5,19] even for gapless fragments.

Levy *et al. *[20] designed a greedy heuristic algorithm that concatenates the fragments with minimum conflicts. The greedy heuristic algorithm is very fast but not very accurate when the error rate of fragments is high. Later, Bansal and Bafna [21] developed a software package HapCUT and the algorithm is based on the idea of building a graph from the sequenced fragments, in which each SNP site corresponds to a node in the graph and two nodes are connected by an edge if there exists a fragment that covers both SNP sites (which correspond to the two nodes). It then tries to minimize the MEC cost of the reconstructed haplotypes by iteratively finding max-cuts in the associated graph. Bansal *et al. *[22] designed a Markov Chain Monte Carlo (MCMC) algorithm, HASH. Both HASH and HapCUT have better performance than the greedy heuristic algorithm proposed in [20].

Recently, He *et al. *[16] gave a dynamic programming algorithm that can give the optimal solution to the haplotype assembly problem with MEC. The time complexity of the algorithm is *O*(*m *× 2*^k ^*× *n*), where *m *is the number of fragments, *n *is the number of SNP sites, and *k *is the length of the longest fragments. This algorithm works well for *k *≤ 15. However, it becomes impractical when *k *is large.

In this paper, we propose a heuristic algorithm for the haplotype assembly problem with MEC. It is worth mentioning that in HapCUT [21] and the dynamic programming algorithm proposed in [16], the authors assumed that the two constructed haplotypes are complementary with each other, i.e. there are only 2 choices at a SNP site in the reconstructed haplotypes. We drop this assumption in our heuristic algorithm. As a result, there are 4 choices at a SNP site in the reconstructed haplotypes. We have tested our algorithm on a set of benchmark datasets and compare it with several state-of-the-art algorithms. Experiments show that our algorithm is highly accurate. It outperforms most of the existing programs when the error rate of input fragments is high.

## Preliminaries

The input to the haplotype assembly problem is a set of fragments sequenced from the two copies of a chromosome of a single individual. Each fragment covers some SNP sites. We assume that all the fragments have been pre-aligned to a reference DNA sequence. As a result, we can organize the input fragments as an *m *× *n *matrix *M *(called *fragment matrix*), where *m *is the number of fragments and *n *is the number of SNP sites. Each row of *M *corresponds to a fragment that can be represented as a string on the alphabet ∑ = {*a*, *c*, *g*, *t*, -}, where '-' indicates a space when the SNP site is not covered by the fragment or the SNP value cannot be determined with enough confidence. The *start *(respectively, *end*) position of a fragment is defined as the first (respectively, last) position in the corresponding row that is not a '-'. In the middle of a fragment, '-'s are allowed due to data missing or paired-end fragments. Throughout the remainder of this paper, we will use the two notations, i.e. fragments and rows of *M *interchangeably when there is no ambiguity. Moreover, we will use columns and SNP sites interchangeably when there is no ambiguity.

**Figure 1:**
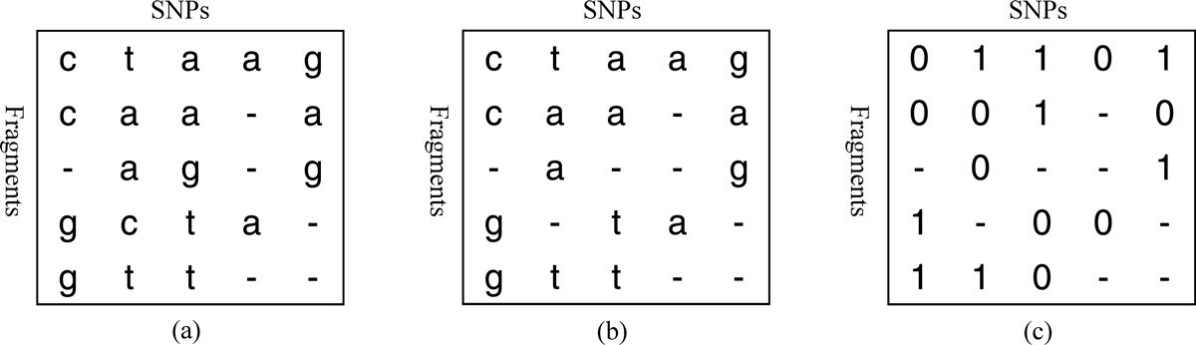
**Illustration of the preprocessing on the input fragment matrix**. (a) The original fragment matrix *M*. (b) The matrix obtained from (a) by removing possible errors. (c) The obtained matrix $M^{\prime }$.

It is accepted that there are at most two distinct nucleotides at a SNP site. We assume that a column with more than two distinct nucleotides in *M *must contain errors. In this case, we keep the two distinct nucleotides that appear the most at this column and replace the rest of them with a '-'. After removing errors, *M *can be converted into $M^{\prime }$, in which each entry is encoded by a character from the alphabet $\sum^{\prime }=\left\{0,1\;-\right\}$. Figure 1(a) gives an example of an original input matrix *M *containing errors in some columns, Figure 1(b) is the matrix after error correction. $M^{\prime }$ is given in Figure 1(c).

We say that row *i *covers column *j *in *M *if *M_i_*_,*j *_is not a '-' or there are two integers *p *and *q *with *p < j < q *such that *M_i_*_,*p *_≠ - and *M_i_*_,*q *_= -. The number of rows covering column *i *in *M *is referred to as the *coverage *of column *i*.

Two rows *p *and *q *in *M *are in *conflict *if there exists a column *j *such that *M_p_*_,*j *_≠ *M_q_*_,*j*_, *M_p_*_,*j *_≠ - and *M_q_*_,*j *_≠ -. Obviously, for error-free data, two rows from the same copy of a chromosome should not conflict with each other, and two rows which conflict with each other must come from different copies of a chromosome. The distance between two rows *i *and *j*, denoted by *D*(*i*, *j*), is defined as the generalized hamming distance as follows:

(1) $$D\left(i,j\right)=\overset{n}{\underset{k=1}{\sum }}d\left(M_{i,k},M_{j,k}\right)$$

where

(2) $$d\left(M_{i,k},M_{j,k}\right)=\left\{\begin{array}{cc} 1 & \text{if}\;M_{i,k}\neq M_{j,k,}M_{i,k}\neq -\text{and}\;M_{j,k}\neq -;\\ 0 & \text{otherwise}\text{.} \end{array}\right)$$

Minimum error correction (MEC) is a commonly used model for the haplotype assembly problem. For the haplotype assembly problem with MEC, the input is a fragment matrix *M*, the task is to partition the fragments in *M *into two groups and construct two haplotypes (*h*_1_, *h*_2_), one from each group, such that the total number of conflicts (errors) between the fragments and the constructed haplotypes (*h*_1_, *h*_2_) is minimized.

## Methods

In this section, we will describe the algorithms used to solve the problem. We first design a dynamic programming algorithm that gives an exact solution and runs in *O*(*n *× 2*^t ^*× *t*) time, where *n *is the number of columns in *M*, and *t *is the maximum coverage of a column in *M*. The dynamic programming algorithm will be very slow when *t *is large. We then design a heuristic algorithm that first computes an initial pair of haplotypes by using the dynamic programming algorithm on only a subset of *M*. This initial pair of haplotypes can be viewed as an approximation to the optimal solution. To obtain a better solution, we further introduce some techniques to refine the initial solution.

### A dynamic programming algorithm

Recall that the goal of the haplotype assembly problem is to partition the rows of the input fragment matrix *M *into two groups, each of which determining a haplotype. To obtain an optimal partition, a naive approach is to enumerate all possible partitions on the rows of *M*, among which we then choose the one minimizing MEC. For an instance with *m *rows, there are 2*^m ^*total partitions, and thus the approach does not work in practice. Here we introduce a dynamic programming algorithm for the haplotype assembly problem with MEC that runs in *O*(*n *× 2*^t ^*× *t*) time, where *n *is the number of columns in *M*, and *t *is the maximum coverage of a column in *M*.

Before we give the details of the dynamic programming algorithm, we first define some basic notations that will be used later:

•*R_i_*: the set of rows covering column *i *in *M*.

•*P_j_*(*i*): the *j*-th partition on *R_i_*.

•*Q_j_*(*i*): the *j*-th partition on *R_i _*∩ *R_i_*_+1_.

•*P_j_*(*i*)*|_Ri_*_∩*Ri*+1_: the partition on *R_i _*∩ *R_i_*_+1 _obtained from *P_j_*(*i*) by restriction on the rows in *R_i _*∩ *R_i_*_+1_.

•*QQ_j_*(*i*): the set of partitions *P_k_*(*i*) such that *P_k_*(*i*)*|_Ri_*_∩*Ri*+1 _= *Q_j_*(*i*).

•*C*(*P_j_*(*i*)): the minimum number of corrections to be made in column *i *of *M *when the partition on *R_i _*is indicated by *P_j_*(*i*).

•*MEC*(*i*, *P_j_*(*i*)): the optimal cost for the first *i *columns in *M *such that column *i *has a partition *P_j_*(*i*).

In order to compute *MEC*(*i *+ 1, *P_j_*(*i *+ 1)) efficiently, we define

(3) $$ME(i,Q_{j}(i))=min_{P_{k}(i)\in QQ_{j}(i)}MEC(i,P_{k}(i)).$$

Let *P_j_*(*i *+ 1) be the *j*-th partition on *R_i_*_+1_, *Q_k_*(*i*) = *P_j_*(*i *+ 1)*|_Ri_*_∩*Ri*+1_. The recursion formula of the dynamic programming algorithm is illustrated as follows:

(4) $$MEC(i+1,P_{j}\left(i+1)\right)=C\left(P_{j}\left(i+1\right)\right)+ME\left(i,Q_{k}\left(i\right)\right).$$

Based on *P_j_*(*i *+ 1), we can get *Q_k_*(*i*) in *O*(*t*) time. Furthermore, we know the majority value (0 or 1) at column (*i *+ 1) in each group. To compute *C*(*P_j_*(*i *+ 1)), we can simply count the number of minorities in each group (at column (*i *+ 1)) separately, and then add them up. Thus, it takes *O*(*t*) time to compute *C*(*P_j_*(*i *+ 1)).

The optimal MEC cost for partitioning all the rows of *M *is the smallest *MEC*(*n*, *P_j_*(*n*)) over all possible *P_j_*(*n*), where *n *is the number of columns in *M*. A standard backtracking process can be used to obtain the optimal solution.

Let us look at the time complexity of the dynamic programming algorithm. To compute each *MEC*(*i *+ 1, *P_j_*(*i *+ 1)) in Equation (4), it requires *O*(*t*) time to compute *C*(*P_j_*(*i *+ 1)). Thus, it takes *O*(*n *× 2*^t ^*× *t*) time to compute all *C*(*P_j_*(*i *+ 1))s for all the *n *columns in *M*. Now, let us look at the way to compute *M E*(*i*, *Q_k_*(*i*))s. For each partition *P_j_*(*i*) on *R_i_*, we can get *Q_k_*(*i*) = *P_j_*(*i*)*|_Ri_*_∩*Ri*+1 _in *O*(*t*) time. We then update *ME*(*i*, *Q_k_*(*i*)) if the current value of *ME*(*i*, *Q_k_*(*i*)) is greater than *MEC*(*i*, *P_j_*(*i*)). There are at most 2*^t ^P_j_*(*i*)s on *R_i_*. Thus, it takes *O*(*t *× 2*^t^*) time to compute all *ME*(*i*, *Q_k_*(*i*))s on *R_i_*. Since there are *n *columns in *M*, the total time required for computing all *ME*(*i*, *Q_k_*(*i*))s is *O*(*n *× 2*^t ^*× *t*).

**Theorem 1 ***Given a fragment matrix M*, *there is an O*(*n *× 2*^t ^*× *t*) *time algorithm to compute an optimal solution for the haplotype assembly problem with MEC*, *where n is the number of columns in M*, *and t is the maximum coverage of a column in M*.

### Obtaining an initial solution via randomized sampling

The dynamic programming algorithm works well when *t *is relatively small. However, it will be very slow when *t *is large. To solve the problem when *t *is large, we look at each column *i *at a time, randomly select a fixed number of rows, say, *boundOfCoverage*, from the set of rows covering it and delete the characters in the remaining rows at all the columns after *i *- 1. After that, the coverage of each column in the newly obtained submatrix is at most *boundOfCoverage*. We then run the dynamic programming algorithm on the submatrix. The resulting pair of haplotypes, which is referred to as the *initial solution*, can be viewed as an approximation to the optimal solution.

The detailed procedure for obtaining a submatrix from *M *via the randomized sampling approach is as follows:

1. Compute the coverage *c_i _*for each column *i *in *M*.

2. For *i *= 1 to *n*, perform the following steps.

3. If *c_i _*≤ *boundOfCoverage*, do nothing and goto the next column. Otherwise, goto step 4.

4. Randomly choose *boundOfCoverage *rows from the set of rows covering column *i*. Let $\bar{S}$ be the set of rows covering column *i *but are not chosen during this process.

5. For each row $r\in \bar{S}$, cut *r *from column *i *such that it no longer covers any column larger than *i *(including *i*). Accordingly, we need to reduce *c_j _*by 1 for each *i *≤ *j *≤ *k*, where *k *is the end position of *r *before being cut.

By employing this randomized sampling strategy, we can always make sure that the maximum coverage is bounded by the threshold *boundOfCoverage *in the selected submatrix. How to choose a proper value for *boundOfCoverage*? Actually, there is a tradeoff between the running time and the quality of the initial solution output by the dynamic programming algorithm. On one hand, reducing *boundOfCoverage *can reduce the running time of the algorithm. However, on the other hand, increasing *boundOfCoverage *can maintain more information from *M*. As a result, the initial solution output by the dynamic programming algorithm has a higher chance to be close to the optimal solution. In practice, *boundOfCoverage *is generally no larger than 15, which is feasible in terms of running time and is large enough to sample sufficient information from *M*. See Section Experiments for a detailed discussion on how the size of *boundOfCoverage *affects the initial solution.

### Refining the initial solution with all fragments

In the newly obtained submatrix, it is possible that (1) some columns are not covered by any rows, thus leaving the haplotype values at these SNP sites undetermined in the initial solution, (2) the haplotype values at some SNP sites in the initial solution are wrongly determined due to the lack of sufficient information sampled from *M *during the randomized sampling process. In this subsection, we try to refine the initial solution with all input fragments, aiming to fill haplotype values that are left undetermined and correct those that are wrongly determined.

The refining procedure contains several iterations. In each iteration, we take two haplotypes as its input and output a new pair of haplotypes. Initially, the two haplotypes in the initial solution are used as the input to the first iteration. The haplotypes output in an iteration are then used as the input to the subsequent iteration. In each iteration, we try to reassign the rows of *M *into two groups based on the two input haplotypes. More specifically, for each row *r *of *M*, we first compute the generalized hamming distance between *r *and the two haplotypes. Then, we assign *r *to the group associated with the haplotype that has the smaller (generalized hamming) distance with *r*. After reassigning all rows of *M *into two groups, we can compute a haplotype from each of the two groups by majority rule. At the same time, we can also obtain the corresponding MEC cost.

The refining procedure stops when, at the end of some iteration, the obtained haplotypes no longer change, or when a certain number of iterations have been finished. The two haplotypes output in the last iteration are the output of the refining procedure.

### Voting procedure

To further reduce the effect of randomness caused by the randomized sampling process, we try to obtain several different submatrices from *M *by repeating the randomized sampling process several times. Accordingly, we can obtain several initial solutions, one derived from each submatrix. Furthermore, we can refine these initial solutions with all fragments. Given a set of solutions, each of which containing a pair of haplotypes, the goal here is to compute a single pair of haplotypes by adopting a voting procedure.

In the voting procedure, the two haplotypes are computed separately. We next see how to compute one of the two haplotypes. The other case is similar. Let *S *be the set of solutions used for voting. First, we find a set of haplotypes (denoted by *S*_1_), one from each solution in *S*, such that the haplotypes in *S*_1 _all correspond to the same copy of a chromosome. With *S*_1_, we can then compute a haplotype by majority rule. Simply speaking, at each SNP site, we count the number of 0s and 1s at the given SNP site over the haplotypes in *S*_1_. If we have more 0s, the resulting haplotype takes 0 at the SNP site, otherwise, it takes 1.

How to find *S*_1_? First, we need to clarify that the two haplotypes in each solution in *S *are unordered. That is, given a solution *H *= (*h*_1_, *h*_2_), we do not know which chromosome copy *h*_1 _(or *h*_2_) corresponds to. So, we should first find the correspondence between the haplotypes in different solutions. Let $H_{1}=\left(h_{1}^{1},h_{2}^{1}\right),.\;.\;.,H_{y}=\left(h_{1}^{y},h_{2}^{y}\right)$ be the set of solutions in *S*. Without loss of generality, assume that the MEC cost associated with *H*_1 _is the smallest among all the *y *solutions. We use *H*_1 _as our reference and try to find the correspondence between haplotypes in *H*_1 _and other solutions. For each *i *(1 *< i *≤ *y*), we first compute two generalized hamming distances $D\left(h_{1}^{1},h_{1}^{i}\right)$ and $D\left(h_{1}^{1},h_{2}^{i}\right)$. If $D\left(h_{1}^{1},h_{1}^{i}\right)<D\left(h_{1}^{1},h_{2}^{i}\right)$, we claim that $h_{1}^{i}$ corresponds to the same chromosome copy as $h_{1}^{1}$. Otherwise, $h_{2}^{i}$ corresponds to the same chromosome copy as $h_{1}^{1}$. As a result, the set of haplotypes in *S *that correspond to the same chromosome copy as $h_{1}^{1}$ is the *S*_1 _we want to find.

Assume that at the beginning of this procedure, we obtain *x *solutions by repeating the randomized sampling process along with the refining procedure *x *times. It is worth mentioning that in the voting procedure, we only use part of the solutions, say, the first *y *(*y *≤ *x*) solutions with the highest quality. Given two solutions *A *and *B*, we say that *A *has *higher quality *than *B *if the MEC cost associated with *A *is smaller than that of *B*. In this case, we assume that *A *is much closer to the optimal solution and contains less noises than *B*. To reduce the sideeffect of noises and improve the quality of the solution output by the voting procedure, it is helpful to use only solutions with high quality in the voting procedure.

### Summarization of the algorithm

Generally speaking, given an input fragment matrix *M*, our heuristic algorithm can be summarized as the following four steps.

Step 1: We first perform a preprocessing on *M *to detect possible errors in it. After removing errors from *M*, we further convert it into $M^{\prime }$ in which each entry is encoded by a character from the alphabet $\sum^{\prime }=\left\{0,1,-\right\}$. See Section Preliminaries for more details. $M^{\prime }$ is used as the input to the following steps.

Step 2: We compute an initial solution by running the dynamic programming algorithm on a subset of $M^{\prime }$. The submatrix is computed by using the randomized sampling approach.

Step 3: Refine the initial solution with all the fragments in $M^{\prime }$, instead of the submatrix that is used to generate the initial solution in Step 2.

Step 4: To further reduce the effect of randomness caused by the randomized sampling process, we repeat Step 2 and Step 3 several times. Each repeat ends with a solution, from which we then compute a single pair of haplotypes by adopting the voting procedure. The resulting pair of haplotypes is the output of our algorithm.

## Results

We have tested our algorithm on a set of benchmark datasets and compare its performance with several other algorithms. The main purpose here is to evaluate how accurately our algorithm can reconstruct haplotypes from input fragments. All the tests have been done on a Windows-XP (32 bits) desktop PC with 3.16 GHz CPU and 4GB RAM.

The benchmark we use was created by Geraci in [23]. It was generated by using real human haplotype data from the HapMap project [4]. There are three parameters associated with the benchmark, i.e *haplotype length*, *error rate *and *coverage rate*, denoted by *l*, *e*, *c*, respectively. Each parameter has several different values, *l *= 100, 350, and 700, *e *= 0.0, 0.1, 0.2 and 0.3, *c *= 3, 5, 8 and 10. Note that unlike the "coverage" defined in Section Preliminaries, the coverage rate *c *defined in this benchmark refers to the number of times each of the two haplotypes replicates when generating the dataset. In other words, given an instance in the benchmark, i.e. a fragment matrix, there are up to 2*c *rows which take non '-' value at each column in the matrix. For each combination of the three parameters, there are 100 instances in the benchmark. As for the details on how to generate the benchmark, the reader is referred to [23].

Throughout our experiments, we measure the performance of our algorithm by the *reconstruction rate*, a frequently used criterion in the haplotype assembly problem. Given a problem instance in the benchmark, the reconstruction rate is defined as follows:

(5) $$R_{\hat{H},H}=1-\frac{min(D^{\prime }(h_{1},{\hat{h}}_{1})+D^{\prime }(h_{2},{\hat{h}}_{2}),D^{\prime }(h_{1},{\hat{h}}_{2})+D^{\prime }(h_{2},{\hat{h}}_{1}))}{2n}$$

where *H *= (*h*_1_, *h*_2_) is the pair of correct haplotypes that is used to generate the problem instance, and is thus known a prior, $Ĥ=\left({ĥ}_{\text{1}},{ĥ}_{\text{2}}\right)$ is the pair of haplotypes output by the algorithm, *n *is the length of the haplotypes, and $D^{\prime }$ is the hamming distance between two haplotypes. More specifically, $D^{\prime }$ is defined as follows:

(6) $$D^{\prime }\left(h_{i},{ĥ}_{j}\right)=\overset{n}{\underset{k=1}{\sum }}d^{\prime }\left(h_{i}\left[k\right],{ĥ}_{j}\left[k\right]\right)$$

where

(7) $$d^{\prime }\left(h_{i}\left[k\right],{ĥ}_{j}\left[k\right]\right)=\left\{\begin{array}{cc} 0 & \text{if}\;h_{i}\left[k\right]={ĥ}_{j}\left[k\right];\\ 1 & \text{otherwise}. \end{array}\right)$$

Intuitively speaking, the reconstruction rate measures the ability of an algorithm to reconstruct the correct haplotypes.

Recall that in Step 2 of our algorithm, we try to compute an initial solution by using only a subset of the input matrix. The initial solution forms the basis for the following steps of our algorithm and is closely related to the parameter *boundOfCoverage*. Briefly speaking, *boundOfCoverage *is the maximum coverage of a column in the submatrix selected during this step. For a formal description of *boundOfCoverage*, we refer you to Section Methods. In this experiment, we first evaluate how the size of *boundOfCoverage *affects the initial solution. As aforementioned earlier, *boundOfCoverage *is generally no larger than 15. Here we consider three different sizes of *boundOfCoverage*, i.e. 10, 12 and 15. Given a problem instance, we can obtain three initial solutions by using the three different sizes of *boundOfCoverage*, respectively. As an example, we choose the set of benchmark datasets with *l *= 350 and *e *= 0.2. For each combination of the coverage rate *c *and *boundOfCoverage*, there are 100 instances and we compute the average of the reconstruction rates over the 100 instances. The results are listed in Table 1.

**Table 1:** Evaluation of how the size of boundOfCoverage affects the initial solution.

	c = 3	c = 5	c = 8	c = 10
10	0.708(*<*0.1)	0.753(*<*0.1)	0.764(0.14)	0.774(0.18)
12	0.728(*<*0.1)	0.785(*<*0.1)	0.794(0.15)	0.797(0.21)
15	0.776(0.30)	0.837(0.33)	0.841(0.36)	0.857(0.45)

There are 3 different sizes of *boundOfCoverage*, i.e. 10, 12 and 15, and 4 different coverage rates *c*, i.e. 3, 5, 8 and 10. The number outside each bracket refers to the average reconstruction rate under the corresponding parameter setting, while the one enclosed in the bracket refers to the average running time in seconds.

From Table 1, we can see that for a fixed coverage rate *c*, when increasing the size of *boundOfCoverage*, the reconstruction rate of the obtained initial solution gets higher, and the running time increases accordingly. Since the reconstruction rate in the case where *boundOfCoverage *= 12 is relatively high, and the running time is feasible, we will set *boundOfCoverage *to be 12 in the following experiments.

Next, to evaluate the performance of our algorithm, we have tested it on the set of benchmark datasets. The parameters we use are as follows: *boundOfCoverage *= 12, *x *= 100, and *y *= 11, where *x *is the number of initial solutions obtained in Step 4, i.e. the number of times we repeat Step 2 and Step 3, and *y *is the number of solutions used for voting in Step 4. The results for *l *= 100, 350 and 700 are given in the last column in Table 2 Table 3 and Table 4, respectively. In [23], Geraci compared the reconstruction rates of seven state-of-the-art algorithms on the same benchmark datasets. These seven algorithms are SpeedHap [24], Fast hare [25], 2d-mec [26], HapCUT [21], MLF [27], SHR-three [28] and DGS, the greedy heuristic proposed in [20]. For a full review of these seven algorithms, the reader is referred to [23]. For the sake of comparison, we list the reconstruction rates of the seven algorithms, see Columns 3 *- *9 in Table 2, Table 3 and Table 4. Note that the results for the seven algorithms are directly taken from [23]. Each reconstruction rate shown in the three tables is the average over 100 instances under the same parameter setting.

**Table 2:** Comparisons of the algorithms when *l *= 100.

*e*	*c*	SpeedHap	Fast Hare	2d-mec	HapCUT	MLF	SHR-three	DGS	Ours
0.0	3	0.999	0.999	0.990	**1.000**	0.973	0.816	**1.000**	1.000
	5	**1.000**	0.999	0.997	**1.000**	0.992	0.861	**1.000**	1.000
	8	**1.000**	**1.000**	**1.000**	**1.000**	0.997	0.912	**1.000**	1.000
	10	**1.000**	**1.000**	**1.000**	**1.000**	0.998	0.944	**1.000**	1.000
0.1	3	0.895	0.919	0.912	0.929	0.889	0.696	**0.930**	0.973
	5	0.967	0.965	0.951	0.920	0.970	0.738	**0.985**	0.996
	8	0.989	**0.993**	0.983	0.901	0.985	0.758	0.989	0.999
	10	0.990	**0.998**	0.988	0.892	0.995	0.762	0.997	1.000
0.2	3	0.623	0.715	0.738	**0.782**	0.725	0.615	0.725	0.903
	5	0.799	0.797	0.793	**0.838**	0.836	0.655	0.813	0.963
	8	0.852	0.881	0.873	0.864	**0.918**	0.681	0.878	0.990
	10	0.865	0.915	0.894	0.871	**0.938**	0.699	0.917	0.996
0.3	3	0.480	0.617	**0.623**	0.602	0.618	0.557	0.611	0.776
	5	0.637	0.639	0.640	0.629	**0.653**	0.599	0.647	0.874
	8	0.667	0.661	0.675	0.673	**0.697**	0.632	0.663	0.950
	10	0.676	0.675	0.678	0.709	**0.715**	0.632	0.688	0.972

The columns *e *and *c *refer to the error rate and coverage rate, respectively. Columns 3-9 represent the reconstruction rate of the seven algorithms, i.e. SpeedHap, Fast Hare, 2d-mec, HapCUT, MLF, SHR-three and DGS. For each combination of *e *and *c*, the best among the seven algorithms is highlighted in bold. The last column lists the reconstruction rate of our algorithm.

**Table 3:** Comparisons of the algorithms when *l *= 350.

e	c	SpeedHap	Fast Hare	2d-mec	HapCUT	MLF	SHR-three	DGS	Ours
0.0	3	0.999	0.990	0.965	**1.000**	0.864	0.830	0.999	1.000
	5	**1.000**	0.999	0.993	**1.000**	0.929	0.829	**1.000**	1.000
	8	**1.000**	**1.000**	0.998	**1.000**	0.969	0.895	**1.000**	1.000
	10	**1.000**	0.999	0.999	**1.000**	0.981	0.878	**1.000**	1.000
0.1	3	0.819	0.871	0.837	**0.930**	0.752	0.682	0.926	0.970
	5	0.959	0.945	0.913	0.913	0.858	0.724	**0.978**	0.993
	8	0.984	0.985	0.964	0.896	0.933	0.742	**0.996**	0.999
	10	0.984	0.995	0.978	0.888	0.962	0.728	**0.998**	1.000
0.2	3	0.439	0.684	0.675	**0.771**	0.642	0.591	0.691	0.877
	5	0.729	0.746	0.728	**0.831**	0.728	0.632	0.769	0.953
	8	0.825	0.853	0.791	**0.862**	0.798	0.670	0.842	0.988
	10	0.855	0.877	0.817	0.867	0.831	0.668	**0.878**	0.994
0.3	3	0.251	0.590	**0.593**	0.565	0.581	0.548	0.578	0.725
	5	0.578	0.602	0.606	0.582	0.606	0.557	**0.609**	0.833
	8	0.629	0.626	0.623	0.621	**0.634**	0.604	0.628	0.922
	10	0.638	0.644	0.634	**0.664**	0.641	0.619	0.641	0.951

The columns *e *and *c *refer to the error rate and coverage rate, respectively. Columns 3-9 represent the reconstruction rate of the seven algorithms, i.e. SpeedHap, Fast Hare, 2d-mec, HapCUT, MLF, SHR-three and DGS. For each combination of *e *and *c*, the best among the seven algorithms is highlighted in bold. The last column lists the reconstruction rate of our algorithm.

**Table 4:** Comparisons of the algorithms when *l *= 700.

e	c	SpeedHap	Fast Hare	2d-mec	HapCUT	MLF	SHR-three	DGS	Ours
0.0	3	0.999	0.988	0.946	**1.000**	0.787	0.781	0.999	0.997
	5	**1.000**	0.999	0.976	**1.000**	0.854	0.832	**1.000**	0.999
	8	**1.000**	**1.000**	0.992	**1.000**	0.919	0.868	**1.000**	1.000
	10	**1.000**	0.999	0.997	**1.000**	0.933	0.898	**1.000**	1.000
0.1	3	0.705	0.829	0.786	0.927	0.698	0.668	**0.931**	0.951
	5	0.947	0.949	0.880	0.916	0.809	0.716	**0.977**	0.989
	8	0.985	0.986	0.948	0.896	0.863	0.743	**0.987**	0.997
	10	0.986	0.995	0.965	0.889	0.884	0.726	**0.997**	0.998
0.2	3	0.199	0.652	0.647	**0.753**	0.624	0.591	0.669	0.837
	5	0.681	0.712	0.697	**0.825**	0.682	0.617	0.741	0.927
	8	0.801	0.808	0.751	**0.856**	0.747	0.653	0.818	0.974
	10	0.813	**0.872**	0.778	0.861	0.765	0.675	0.861	0.982
0.3	3	0.095	0.581	**0.583**	0.552	0.570	0.536	0.573	0.676
	5	0.523	0.591	**0.596**	0.555	0.594	0.562	0.595	0.777
	8	**0.616**	0.615	0.613	0.597	0.614	0.611	0.614	0.876
	10	0.627	0.616	0.622	**0.645**	0.625	0.625	0.622	0.909

The columns *e *and *c *refer to the error rate and coverage rate, respectively. Columns 3-9 represent the reconstruction rate of the seven algorithms, i.e. SpeedHap, Fast Hare, 2d-mec, HapCUT, MLF, SHR-three and DGS. For each combination of *e *and *c*, the best among the seven algorithms is highlighted in bold. The last column lists the reconstruction rate of our algorithm.

Take a close look at the three tables, we can see that (1) each of the seven algorithms studied in [23] only works well in some cases, e.g., SpeedHap works well when the error rate is low (≤ 0.1), while MLF works well when the error rate is high (≥ 0.2); (2) the reconstruction rates of all the seven algorithms are relatively low when the error rate of input fragments is high. For example, in the case where *l *= 700, *e *= 0.3 and *c *= 10, the best reconstruction rate of the seven other algorithm is 0.645. Compared with its competitors, our algorithm can give solutions with high reconstruction rate. It outperforms its competitors in almost all cases, especially in cases in which the error rate of fragments is high (≥ 0.2). We also notice that when the error rate is 0, our algorithm may introduce some errors in the output solution, e.g., in the case where *l *= 700, *c *= 3, *e *= 0.0. However, even in this case, the reconstruction rate can still reach up to 0.997.

## Discussion

In the first step of our algorithm, we perform a preprocessing on the input fragment matrix. This allows us to detect errors in the input. For example, for the benchmark datasets with *l *= 350 and *e *= 0.3, Step 1 of our algorithm can identify about 47%, 55%, 59% and 61% of the total errors for the cases *c *= 3, 5, 8 and 10, respectively. Thus, Step 1 has significant importance to the following steps of our algorithm.

**Figure 2:**
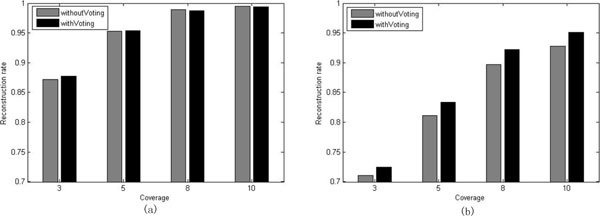
**Illustration of the effect of the voting procedure**. The reconstruction rates for the final version of our algorithm and the one without the voting procedure are depicted by black and gray bar, respectively. The error rate for the benchmark used in (a)(respectively, (b)) is 0.2 (respectively, 0.3).

Next, we further investigate how the voting procedure in Step 4 affects the performance of our algorithm. In Step 4, we first obtain *x *solutions, from which we then choose the first *y *(*y *≤ *x*) solutions with the smallest MEC cost. The *y *solutions are then used in the voting procedure to compute the final solution of our algorithm. To demonstrate the effect of the voting procedure, we compare the final version of our algorithm with the one without the voting procedure. For the version without the voting procedure, we simply outputs the solution with the smallest MEC cost among all the *x *solutions in Step 4. As an example, we have tested both versions on the set of benchmark datasets with *l *= 350. The parameters are as follows: *boundOfCoverage *= 12, *x *= 100 and *y *= 11. Figure 2(a) (respectively, 2(b)) shows the results for the two versions in the case where *e *= 0.2 (respectively, *e *= 0.3). The results for *e *= 0.0 and 0.1 are similar as that of *e *= 0.2, and we omit it here.

From Figure 2(a), we can see that the two versions of our algorithm have almost the same reconstruction rate. However, when *e *= 0.3, the final version of our algorithm has higher reconstruction rate than the one without the voting procedure, see Figure 2(b). Thus, the voting procedure is essential for our algorithm.

**Figure 3:**
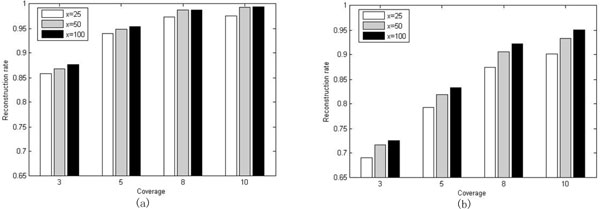
**Evaluation of how the size of ***x ***affects the performance of our algorithm**. The reconstruction rates for *x *= 25, 50 and 100 are depicted by white, gray and black bar, respectively. The error rate for the benchmark used in (a)(respectively, (b)) is 0.2 (respectively, 0.3).

To see how the size of the parameter *x *affects the reconstruction rate of our algorithm, we have tested our algorithm with three different sizes of *x*, i.e. 25, 50, 100. The values of *boundOfCoverage *and *y *are fixed to be 12 and 11, respectively. The tests are done on the set of benchmark datasets with *l *= 350. The results for *e *= 0.2 and 0.3 are shown in Figure 3(a) and 3(b), respectively. For *e *= 0.0 and 0.1, the reconstruction rates are almost the same in all the three cases, and we do not list it here. As can be seen from Figure 3(a), the reconstruction rate increases with the increasing of *x *in the cases where *c *= 3 and 5. For *c *= 8 and 10, the cases where *x *= 50 and 100 have almost the same reconstruction rate which is higher than that in the case where *x *= 25. As for Figure 3(b), it is much more obvious that the reconstruction rate increases as *x *gets larger.

## Conclusion

In this paper, we propose a heuristic algorithm for the haplotype assembly problem. Experiments show that our algorithm is highly accurate. It outperforms most of the existing programs when the error rate of input fragments is high.

## Competing interests

The authors declare that they have no competing interests.

## Authors' contributions

FD participated in the design of the study, performed the experiments and drafted the manuscript. WC participated in the design of the study and helped to draft the manuscript. LW conceived the study, participated in its design and helped to draft the manuscript. All authors read and approved the final manuscript.

## Declarations

The publication costs for this article were funded by the corresponding author's institution.

## References

[B1] Hoehe M Köpke K Wendel B Rohde K Flachmeier C Kidd K Berrettini W Church G Sequence variability and candidate gene analysis in complex disease: association of *μ *opioid receptor gene variation with substance dependence Human Molecular Genetics 2000 9 19 2895 2908 10.1093/hmg/9.19.2895 11092766

[B2] Schaid D Evaluating associations of haplotypes with traits Genetic epidemiology 2004 27 4 348 364 10.1002/gepi.20037 15543638

[B3] Bonizzoni P Della Vedova G Dondi R Li J The haplotyping problem: an overview of computational models and solutions Journal of Computer Science and Technology 2003 18 6 675 688 10.1007/BF02945456

[B4] Altshuler D Wing T A haplotype map of the human genome Nature 2005 437 7063 1299 1320 10.1038/nature04226 16255080 PMC1880871

[B5] Cilibrasi R Van Iersel L Kelk S Tromp J On the complexity of several haplotyping problems Algorithms in Bioinformatics 2005 128 139

[B6] Schwartz R Theory and algorithms for the haplotype assembly problem Communications in Information and Systems 2010 10 23 38

[B7] Zhang X Wang R Wu L Chen L Models and algorithms for haplotyping problem Current Bioinformatics 2006 1 104 114

[B8] Gusfield D Haplotyping as perfect phylogeny: conceptual framework and efficient solutions Proceedings of the Sixth Annual International Conference on Computational Biology 2002 ACM 166 175

[B9] Wang L Xu Y Haplotype inference by maximum parsimony Bioinformatics 2003 19 14 1773 1780 10.1093/bioinformatics/btg239 14512348

[B10] Stephens M Smith N Donnelly P A new statistical method for haplotype reconstruction from population data The American Journal of Human Genetics 2001 68 4 978 989 10.1086/319501 PMC1275651 11254454

[B11] Halperin E Eskin E Haplotype reconstruction from genotype data using imperfect phylogeny Bioinformatics 2004 20 12 1842 1849 10.1093/bioinformatics/bth149 14988101

[B12] Duitama J Huebsch T McEwen G Suk E Hoehe M ReFHap: A reliable and fast algorithm for single individual haplotyping Proceedings of the First ACM International Conference on Bioinformatics and Computational Biology 2010 160 169 ACM

[B13] Li L Kim J Waterman M Haplotype reconstruction from SNP alignment Journal of Computational Biology 2004 11 2-3 505 516 10.1089/1066527041410454 15285905

[B14] Xie M Wang J Chen J A model of higher accuracy for the individual haplotyping problem based on weighted SNP fragments and genotype with errors Bioinformatics 2008 24 13 i105 i113 10.1093/bioinformatics/btn147 18586702 PMC2718625

[B15] Wang R Wu L Li Z Zhang X Haplotype reconstruction from SNP fragments by minimum error correction Bioinformatics 2005 21 10 2456 2462 10.1093/bioinformatics/bti352 15731204

[B16] He D Choi A Pipatsrisawat K Darwiche A Eskin E Optimal algorithms for haplotype assembly from whole-genome sequence data Bioinformatics 2010 26 12 i183 i190 10.1093/bioinformatics/btq215 20529904 PMC2881399

[B17] Lancia G Bafna V Istrail S Lippert R Schwartz R SNPs Problems, Complexity, and Algorithms Proceedings of the 9th Annual European Symposium on Algorithms 2001 Springer-Verlag 182 193

[B18] Rizzi R Bafna V Istrail S Lancia G Practical Algorithms and Fixed-Parameter Tractability for the Single Individual SNP Haplotyping Problem Workshop on Algorithms in Bioinformatics 2002 Springer 29 43

[B19] Lippert R Schwartz R Lancia G Istrail S Algorithmic strategies for the single nucleotide polymorphism haplotype assembly problem Briefings in bioinformatics 2002 3 23 31 10.1093/bib/3.1.23 12002221

[B20] Levy S Sutton G Ng P Feuk L Halpern A Walenz B Axelrod N Huang J Kirkness E Denisov G The diploid genome sequence of an individual human PLoS biology 2007 5 10 e254 10.1371/journal.pbio.0050254 17803354 PMC1964779

[B21] Bansal V Bafna V HapCUT: an efficient and accurate algorithm for the haplotype assembly problem Bioinformatics 2008 24 16 i153 i159 10.1093/bioinformatics/btn298 18689818

[B22] Bansal V Halpern A Axelrod N Bafna V An MCMC algorithm for haplotype assembly from whole-genome sequence data Genome research 2008 18 8 1336 1346 10.1101/gr.077065.108 18676820 PMC2493424

[B23] Geraci F A comparison of several algorithms for the single individual SNP haplotyping reconstruction problem Bioinformatics 2010 26 18 2217 2225 10.1093/bioinformatics/btq411 20624781 PMC2935405

[B24] Genovese L Geraci F Pellegrini M SpeedHap: an accurate heuristic for the single individual SNP haplotyping problem with many gaps, high reading error rate and low coverage IEEE/ACM Transactions on Computational Biology and Bioinformatics 2008 5 4 492 502 18989037 10.1109/TCBB.2008.67

[B25] Panconesi A Sozio M Fast hare: A fast heuristic for single individual SNP haplotype reconstruction Algorithms in Bioinformatics 2004 266 277

[B26] Wang Y Feng E Wang R A clustering algorithm based on two distance functions for MEC model Computational biology and chemistry 2007 31 2 148 150 10.1016/j.compbiolchem.2007.02.001 17363329

[B27] Zhao Y Wu L Zhang J Wang R Zhang X Haplotype assembly from aligned weighted SNP fragments Computational Biology and Chemistry 2005 29 4 281 287 10.1016/j.compbiolchem.2005.05.001 16051522

[B28] Chen Z Fu B Schweller R Yang B Zhao Z Zhu B Linear time probabilistic algorithms for the singular haplotype reconstruction problem from SNP fragments Journal of Computational Biology 2008 15 5 535 546 10.1089/cmb.2008.0003 18549306

